# Residential environment and breast cancer incidence and mortality: a systematic review and meta-analysis

**DOI:** 10.1186/s12885-015-1098-z

**Published:** 2015-03-28

**Authors:** Tomi F Akinyemiju, Jeanine M Genkinger, Maggie Farhat, Adrienne Wilson, Tiffany L Gary-Webb, Parisa Tehranifar

**Affiliations:** 1 Department of Epidemiology, Columbia University Mailman School of Public Health, New York, NY USA; 2 Herbert Irving Comprehensive Cancer Center, Columbia University Medical Center, New York, NY USA; 3 Department of Health Behavior and Health Education, University of Michigan School of Public Health, Ann Arbor, MI USA; 4 Departments of Community and Behavioral Sciences and Epidemiology, University of Pittsburgh Graduate School of Public Health, Pittsburgh, PA USA

**Keywords:** Breast cancer epidemiology, Residential environment, Socio-economic status, Mortality, Urbanization

## Abstract

**Background:**

Factors beyond the individual level such as those characterizing the residential environment may be important to breast cancer outcomes. We provide a systematic review and results of meta-analysis of the published empirical literature on the associations between breast cancer risk and mortality and features of the residential environment.

**Methods:**

Using PRISMA guidelines, we searched four electronic databases and manually searched the references of selected articles for studies that were published before June 2013. We selected English language articles that presented data on adult breast cancer incidence or mortality in relation to at least one area-based residential (ABR) independent variable.

**Results:**

We reviewed 31 eligible studies, and observed variations in ABR construct definition and measurement, study design, and analytic approach. The most common ABR measures were indicators of socioeconomic status (SES) (e.g., income, education, summary measures of several SES indicators or composite SES). We observed positive associations between breast cancer incidence and urbanization (Pooled RR for urban vs. rural: 1.09. 95% CI: 1.01, 1.19), ABR income (Pooled RR for highest vs. lowest ABR income: 1.17, 95% CI: 1.15, 1.19) and ABR composite SES (Pooled RR for highest vs. lowest ABR composite SES: 1.25, 95% CI: 1.08, 1.44). We did not observe consistent associations between any ABR measures and breast cancer mortality.

**Conclusions:**

The findings suggest modest positive associations between urbanization and residential area socioeconomic environment and breast cancer incidence. Further studies should address conceptual and methodological gaps in the current publications to enable inference regarding the influence of the residential environment on breast cancer.

## Background

Research on breast cancer epidemiology has traditionally focused on investigating genetic, biomedical and individual-level behavioral factors. However, in the last several decades, researchers have begun to also consider the role of the environment in which individuals reside (residential environment). The residential environment as a determinant of health was highlighted in the 1979 Surgeon General report as part of a comprehensive approach to disease prevention [1], and research in this area further intensified following the 2010 Healthy people report [2].

The residential environment may play a role in breast cancer incidence and mortality through the geographic distribution of breast cancer risk factors, access to quality and timely healthcare resources and medical treatment, as well as through psychosocial pathways involving stress and social support [3-5]. For example, parity, lack of breastfeeding and increased alcohol use are associated with area level characteristics such as neighborhood poverty and access to healthcare [6-9]. The residential environment may also promote and/or hinder utilization of or access to early detection and treatment services [10,11], thereby affecting breast cancer mortality and survival. For instance, access to routine screening such as mammography facilities increases the chance that cancer is detected at early stages for which treatment is most effective, and access to healthcare increases the likelihood of adequate treatment [12,13]. Thus, understanding the association between features of the residential environment and breast cancer outcomes may provide insight into factors relevant to risk reduction, adequate screening and timely treatment, and guide primary, secondary and tertiary prevention efforts.

Different aspects of the residential environment are captured through area-based residential (ABR) measures, most often constructed by aggregating or mathematically summarizing the characteristics of individuals residing within an area (e.g., proportion of residents living below federally defined poverty, mean income level of residents in an area). Although these ABR measures may be used as proxies for individual-level factors when such information is lacking, they may also indicate features of residential environment that are associated with an outcome differently or independently of individual-level factors. ABR measures may also be based on the properties of an area that do not simply summarize characteristics of individuals, and thus, have no equivalent measure at the individual level (e.g., population density or urbanization) [14]. Multiple studies have assessed ABR measures in relation to breast cancer incidence and outcomes. However, to our knowledge, no review of the literature on ABR measures and breast cancer has been previously published. The purpose of this review is to: 1) provide a comprehensive synthesis of the published literature on the associations between features of residential environments, measured at the area level, and breast cancer incidence and mortality; and 2) conduct meta-analysis of results, as appropriate. Additionally, we will describe commonalities and differences in the research findings across the two breast cancer outcomes and across racial/ethnic populations, and identify gaps in the literature.

## Methods for evidence acquisition and synthesis

### Search strategy

We employed established PRISMA guidelines for conducting systematic reviews in health [15]. Given that search terms are not fully developed or systematically used, we chose a broad strategy by searching for many general key words in multiple electronic databases. We also used search terms based on previously published studies and added other relevant terms as appropriate. We searched the electronic databases of PubMed, CINAHL, PsychInfo and Web of Science (WOS) using the term “breast cancer” and any of the following key words: neighborhood, neighbourhood, county, census, residential, residence, area-based, geograph*, environment*, walk*, multilevel, multi-level, context*, hierarchical, community. We limited our search to studies of adult human subjects that were published in English. The search period for article inclusion was from database inception to June 30, 2013.

### Eligibility criteria

Eligible articles met all of the following criteria: 1) were published in English; 2) reported results from analysis of original data (including population-based cancer registries); 3) used at least one area-based residential measure as an independent variable in analysis, including both compositional, aggregated (based on characteristics or the aggregation of characteristics of individuals residing in an area) and contextual measures (characteristics of a defined geographic area); 4) used at least one individual-level covariate in addition to an ABR variable; and 5) evaluated female invasive breast cancer incidence/risk or mortality as the outcome. Studies that only examined trends over time, mortality among individuals with breast cancer, and ecological data (i.e., aggregated vs. individual-level outcome data) were excluded.

### Selection strategy

**Figure 1:**
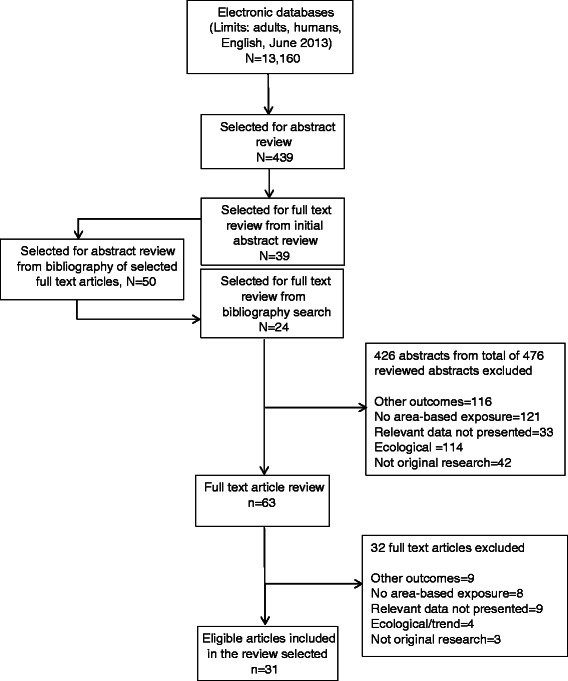
**Publication search and selection results.**

Two authors (PT, TA) independently reviewed study titles, abstracts and full text articles. We reviewed abstracts for study titles selected by at least one reviewer, and reviewed abstracts and full text articles that were selected by both reviewers. Another author (TGW) adjudicated when consensus could not be reached. Figure 1 presents a flowchart of the study selection process and results. We reviewed study titles for 13,160 articles that were identified through the previously mentioned search strategy, and selected 439 articles for the review of the abstracts. Most of the excluded articles either assessed non-cancer outcomes, cancers other than breast cancer, or outcomes such as stage of presentation or treatment, examined trends over time, or compared geographic areas without assessing a specific ABR measure. We selected 39 articles for full text review from the reviewed abstracts. We reviewed 50 additional abstracts identified through manual review of the references of the 39 selected full text articles, and selected an additional 24 articles for full text review. Of the total 63 full text articles reviewed, 31 articles were eligible for data abstraction, and 32 articles were excluded as they examined non-eligible outcomes (e.g., other cancer sites, survival in breast cancer cases; n = 9), lacked any area-based independent (exposure) variable (n = 8), did not present relevant data (n = 8), did not involve original research (n = 3), or presented only ecological results (n = 4).

### Data extraction and synthesis

One author (MF) abstracted data from the selected articles into an electronic database, and two authors (PT, TA) independently verified the coded information against the original articles. All three abstractors met to resolve any inconsistencies by consensus. We extracted data on study characteristics and relevant results for all ABR measures. The study characteristics included the country and region of the study, study design, sample size, data sources, measurement of the residential factors, and age and racial/ethnic distribution if reported. We also retrieved information on the main statistical methods and covariates. Finally, for the extreme two levels of categorical ABR measures (e.g., highest and lowest income levels), we extracted measures of frequency (e.g., rates), or relative measures of association (e.g., relative rate [RR], odds ratio [OR], hazards rate ratios [HR]) and 95% confidence interval (CI), and p-values for linear trend where available.

### Statistical analysis

When measures of association were not presented in the manuscript, we calculated rate ratios using reported age-adjusted rates comparing the highest to the lowest category of each ABR measure [4,16-23]; otherwise, ratio measures were presented for the contrast reported in the original articles. If rates were stratified (e.g. by race/ethnicity), we calculated the rate ratios for each stratum. Due to our interest in understanding racial differences in associations between ABR variables and breast cancer, we present data for un-stratified associations as well as race-stratified associations. If only stratified results were reported, we present rate ratios for the first stratification level.

To be eligible for inclusion in meta-analysis, we required the same ABR construct in at least 2 studies in relation to the same outcome (i.e., incidence/risk or mortality) within the same stratification level, and the studies needed to have sufficient data to calculate a risk estimate and standard error or confidence intervals. We re-calculated the estimates presented in some of the articles to correspond to the same comparison (e.g., estimates presented comparing the lowest to highest income category were re-calculated for the contrast to correspond to the highest versus lowest income category). Based on these criteria, the ABR constructs included in the meta-analysis were ABR measures of education, income, poverty, composite SES and urbanization in relation to breast cancer incidence, and urbanization in relation to breast cancer mortality. If multiple studies presented results that were based on the same dataset for the study period and ABR construct were the same, the study with the larger sample size was included in the meta-analysis. We estimated summary rate ratios comparing the two extreme categories of ABR measures in relation to breast cancer incidence using random-effects models [24]. We calculated the Q-statistic to test for between-studies heterogeneity, and used the I^2^ statistic to calculate the proportion of variation between studies due to heterogeneity. We assessed potential publication bias via inspection of funnel plots and Egger’s test for small-study effects. As the results of the funnel plots and Egger’s test were consistent, we only present the p-values of the Egger’s test for the meta-analysis. We conducted sensitivity analyses of the meta-analysis results when more than two studies were available (influence analysis), and when more than 4 studies were available (meta-regression [25]). All statistical analyses were performed using STATA version 12.0 (Stata Corp, College Station, Texas USA).

## Results

Of the 31 articles that fulfilled our selection criteria [4,16-23,26-47], 24 examined breast cancer incidence or risk only [16-21,27,28,30-35,37,39-47], four examined breast cancer mortality only [26,29,36,38], and three articles examined both incidence/risk and mortality [4,22,23] (Table 1). The number of published articles increased steadily over the past several decades, with only one article in each decade of the 1970s [26] and 1980s [17], 9 articles in the 1990s [16,20-23,30,33,34,39],11 in the 2000s [4,18,19,28,32,35,40-42,46,47], and 9 in 2010 through June 2013 [27,29,31,36-38,43,45,47]. About 75% (n = 23) of the published articles were based in the United States (U.S.), including one article that examined data from both the U.S. and Canada [4,16-21,26,28,31-37,39-43,46,47]; an additional two articles were based in Canada [27,30]. Of the remaining articles, two were conducted in Australia [23,29], two in the United Kingdom (U.K.) [22,44], one in Italy [45] and one in Switzerland [38]. Detailed descriptions of each article and sample characteristics are presented in Table 2.

**Table 1:** **Summary description of studies**

	Total number of studies	Number of studies by breast cancer outcome^Ψ^	
	(n=31)	Incidence/risk	Mortality
		(n=27)	(n=7)
**Publication years**			
2010-2013*	9	6	3
2000-2009	11	11	1
1990-1999	9	9	2
1980-1989	1	1	0
1970-1979	1	0	1
**Study design**			
Cross-sectional	25	23	5
Longitudinal	4	2	2
Case–control	2	2	0
**Country**			
U.S.±	23	21	3
Canada±	3	3	0
U.K.	2	2	1
Australia	2	1	2
Italy	1	1	0
Switzerland	1	0	1
**Geographic unit**			
Census tract	8	8	0
Census block group	8	8	0
County	5	4	3
Zip/Postal code	3	3	0
Other	7	9	4
**Racial composition**			
White/European	11	10	1
African American/Black	9	8	1
Hispanic	7	7	0
Asian/Pacific Islanders	5	5	0
American Indian/Native Alaskan	1	1	0
Other	2	2	0
No data	18	16	6

^*^Publications assessed until June 2013.

^Ψ^3 publications assessed both breast cancer incidence and mortality outcomes.

±1 publication was conducted in the US and Canada.

**Table 2:** **Characteristics of studies of residential environment and breast cancer risk or incidence**

Author, year (location)	Individual-level data source; area level data source	Study design and sample characteristics	Geographic location and unit	Main area based measures (measurement)	Outcome
[26]Blot, 1977 (United States)	NIH publication on US cancer mortality by county; 1960 US Census	Cross-sectional; ≥ 20 years old	Contiguous US; county	Income (Median family income, categorized into 2 groups: <50%, >50% by region and population-size)	Mortality
[17]Devesa, 1980 (United States)	Third national cancer survey 1969–1971; US Census 1970	Cross-sectional Females ≥ 15 years; n=20,914 cases; 92.5% white, *7.5*% black	18 US Standard Metropolitan Statistical Areas; Census Tracts	Education (Median years of education categorized into 5 groups for Whites: <10, 10–10.9, 11–11.9, 12–12.9, and ≥13 years; categorized into 3 groups for blacks: <10, 10–10.9, and ≥11 years) Income (Median family income categorized into 5 groups for Whites: <$9,000, $9,000-10,999, $11,000-12,999, $13,000-14,999, ≥$15,000; and categorized into 3 groups for blacks: <$5,000, $5000-6,999, ≥$7,000)	Incidence
[20]Krieger, 1990 (United States)	SEER 1979–1981; US Census 1980	Cross-sectional; Females; n=4,454 cases; 86% white, 9% black, 5% Hispanic	San Francisco Bay Area; Census block group	Occupational class composition (% employed in “working class” occupations, categorized into 2 groups ≤ 66% and >66% in working class occupations); Poverty (% living below poverty, categorized into two groups (≥20% and < 20%)	Incidence
[16]Baquet, 1991 (United States)	SEER 1978–1982; US Census 1980	Cross-sectional; Females; ≥ 25 years old	San Francisco/Oakland, Atlanta, Detroit; Census tract	Education (Median years of education, categorized into 4 groups: <high school, high school graduates, some college, at least 4 years of college) Income (Median family income, categorized into 4 groups: <$15,000, $15,000-24,999, $25,000-29,999, ≥$30,000)	Incidence
[23]Williams, 1991 (Australia)	Victorian Cancer Registry 1982–1983 Melbourne statistical division 1979–1983; Australian Census 1981	Cross-sectional; Females; 40–74 years old	Melbourne; Local government area	Composite SES (Based on occupational status, income, educational attainment, family instability, persons living in low standard housing likely to have difficulty with English, categorized into deciles)	Incidence mortality
[21]Nasca, 1992 (United States)	New York State Cancer Registry 1978–1982; US Census 1980	Cross-sectional	New York state exclusive of New York City; Minor civil divisions	Urbanization (Population density: [persons/square miles], categorized into quinitles	Incidence
[22]Pollock, 1997 (United Kingdom)	The Thames Cancer Registry 1987–1992; UK Census 1991	Cross-sectional; Females; 40–99 years old; n=22,399 cases	South Thames; Enumeration district	Composite SES “Townsend Index of Social Deprivation” (based on % unemployed, % private household lacking a car, % private household not owner occupied, % private household subject to overcrowding; categorized into deciles)	Incidence mortality
[30]Gorey, 1998 (Canada)	Ontario cancer registry 1986–1993; Canadian Census 1991	Cross-sectional; Females; ≥ 25 years old; n=1,3227 cases	Metropolitan Toronto; Census tract	Poverty (annual household income adjusted for household size, categorized into low (≥23% households below low-income cutoff) and high (<7% of households below criterion))	Incidence
[34]Liu, 1998 (United States)	The Los Angeles County Surveillance Program 1979–1992; US Census 1970, 1980, 1990	Cross-sectional; Females; ≥ 15 years old; n=82,453 cases; 77.9% white, 9.1% black, 9.0% Hispanic, 3.2% Asian, <1% other	Los Angeles County; Census tract	Composite SES (Based on weighted average educational attainment and median household income; categorized into quintiles)	Incidence
[39]Prehn, 1998 (United States)	Northern California cancer center’s greater Bay Area cancer registry 1988–1992; US Census 1990	Cross-sectional; Females; n=22,757 cases; 100% white	San Francisco Bay Area and 20 counties from adjoining regions; Census block group	Education (% with college education, categorized into 2 groups: ≥45% vs. <45%); Income (Median household income, categorized into 2 groups ≥ $50000 and <$50000); Occupational Class (% employed in working class occupations, categorized into 2 groups ≤50% and >50% working class); Poverty (% below poverty level, categorized into 2 groups ≤5% vs.>5%)	Incidence
[33]Krieger, 1999 (United States)	Population-based cancer registry 1988–1992; US Census 1990	Cross-sectional; Females; n=16,120 cases; 78% white, 7% black, 7% Hispanic, 8% Asian	San Francisco Bay Area; Census block group	Composite SES (Combination of occupational class (% employed in “working class” and “professional” occupations) and poverty (% below poverty level); categorized into 3 groups: 1) professional (non-poor and poor), 2) working class, non-poor, 3) working class, poor)	Incidence
[35]Mackillop, 2000 (Canada and United States)	Ontario Cancer Registry (1989–1993); SEER 1988–1992; Canadian Census 1991 and US Census 1990	Cross-sectional	Ontario, Canada; 9 SEER regions in US; Enumeration area in Canada and census tract in the U.S.	Income (Median household income, categorized into deciles. Race-specific deciles in the US for secondary analysis) Natural log of relative income for regression	Incidence
[40]Reynolds, 2004 (United States)	The California Teachers Study cohort with annual linkage to the California Cancer Registry, baseline in 1995 with follow up through Dec 1999; US Census 1990	Prospective cohort; Females; 21–108 years at baseline; n=114,927	California; region	Urbanization (a priori specification of urban counties, categorized into San Francisco Bay area, Southern Coastal area, rest of California)	Incidence
[42]Robert, 2004 (United States)	Population-based case control study 1988–1995; US Census 1990	Case–control; Females; 20–79 years old; n=7,179 cases, 7,488 controls	Wisconsin; Census tract and Zip code	Composite SES (Based on median income, % adults below poverty, % unemployed, % college graduate, categorized into quintiles) Urbanization (Residence in census-defined “urban areas”, categorized into 3 groups: 100% rural, mixed rural/urban, 100% urban)	Risk
[18]Hall, 2005 (United States)	North Carolina State Registry 1995–1999; US Census 1990	Cross-sectional; Females; 27,989 cases, 82% white, 18% non-white	North Carolina; County	Metropolitan areas (Urban Influence Code based on by adjacency or non-adjacency to a Metropolitan Area, and size of the largest communities, categorized into 3 groups- metropolitan; non-metropolitan adjacent to metropolitan; non-metropolitan, nonadjacent to metropolitan areas)	Incidence
[41]Reynolds, 2005 (United States)	The California Cancer Registry 1988–1997; US Census 1990	Cross-sectional; Females; ≥20 years old; 176,302 cases	California; Block group	Composite SES (Based on % with college degree, median family income and % employed in managerial/professional occupations, categorized into quartiles) Urbanization (Population size and density, categorized into 4 groups -urban suburban, city, small town/rural)	Incidence
[32]Krieger, 2006 (United States)	Northern California Cancer Center’s San Franciso; Oakland SEER Registry, Los Angeles Surveillance program, Massachusetts Cancer registry 1978–1982, 1988–1992, 1998-2002/ US Census 1980, 1990, 2000	Cross-sectional; Females; 154,083 cases	San Francisco/ Oakland, Los Angeles county, Massachusetts; Census tract	Composite SES (% below poverty level and % high income ho'useholds (defined as ≥4 times the US median household income), categorized into 5 groups: 1) <5% poverty-<10% high income, 2) <5% poverty-≥10% high income, 3) 5-9% poverty, 4) 10-19% poverty, 5) ≥20% poverty)	Incidence
[44]Shack, 2008 (United Kingdom)	English cancer registries 1998–2003; UK Census 2001 and government databases	Cross-sectional; Females; 210,020 cases	8 UK cancer registries; Postal code of residence	Income deprivation (Based on the income domain of the Index of Multiple Deprivation, categorized into quintiles)	Incidence
[46]Webster, 2008 (United States)	The Massachusetts Cancer Registry 1987–1993; US Census 1980, 1990	Case–control; Females; 548 cases, 490 controls	Cape Cod, Massachusetts; Census block group	Composite SES (Based on median income, % adults below poverty, % unemployed, % college graduate, categorized into quintiles) Poverty (% of adults below poverty level, categorized into 3 groups based on the 20th and 80th percentiles of control women)	Risk
[28]Clegg, 2009 (United States)	National Longitudinal Mortality Study and SEER, 1973–2001; US Census 1970, 1980, 1990	Cross-sectional; Females; ≥ 25 years old; 1739 cases; 78% white, 7% black, 4% Mexican, 1% other Hispanic, 4% Asian/Pacific Islander, 2% other	11 SEER regions	Urbanization (Census definition of urban/rural)	Incidence
[4] Harper, 2009 (United States)	SEER 1987–2004; US Census 1990	Cross-sectional	SEER regions; County	Poverty (% below poverty level, categorized into 4 groups: <10%, 10-14%, 15-19%, ≥20%)	Incidence mortality
[19]Hausaer, 2009 (United States)	NAACCR Registries 1997–2004; USDA 2003	Cross-sectional; Females; 50–74 years old; 587,408 cases; 100% white	29 population-based cancer registries in the North American Association of Central Cancer Registries (NAACCR); County	Poverty (% below poverty level, categorized into 3 groups: <10%, 10-19%, ≥20%) Urbanization (US Dept. of Agriculture codes and population size, categorized into urban, suburban and rural areas)	Incidence
[29]Dobson, 2010 (Australia)	Australian Longitudinal Study on Women’s Health, baseline survey in 1996 with follow up through 2006; The Australian Standard Geographic Classification	Longitudinal; Females; 70–75 years at baseline; 12,400 with 2,803 breast cancer deaths	Australia	Area of residence (Road distance to the closest service center, a measure of population size)	Mortality
[31]Keegan, 2010 (United States)	The California Cancer Registry 1988–2004; US Census 2000	Cross-sectional; Females; 12,563 cases; 100% Hispanic	California/ Cross-sectional; Block groups averaged over census tracts (for SES)	Composite Hispanic Enclave (Based on % linguistically isolated overall and who speak Spanish, speak limited English, speak limited English and speak Spanish, % recent immigrants, % Hispanic, % foreign-born) Composite SES (Based on income, occupation, and housing costs, categorized into quintiles) Combined SES and Hispanic Enclave (Combination of SES and Hispanic enclave, categorized into 4 groups: low SES-high enclave, high SES-low enclave, low SES-low enclave, high SES-high enclave)	Incidence
[45]Spadea, 2010 (Italy)	The Turin Longitudinal Study and the Piedmont Cancer Registry, 1985–1999; Italian Census 1971	Cross-sectional; Females; 30–84 years old; 9,203 cases	Turin, Italy; Census tract	Composite SES (Based on % manual workers, % with low education, % tenants, % living % in houses without bath, % families with a single parent with children, and a crowding index, categorized into quintiles) Relative Index of Inequality (Ratio of regression-based rates for extreme points of the social hierarchy)	Incidence/Risk
[27]Borugian, 2011 (Canada)	The Canadian Cancer Registry 1992–2004; Canadian Census 1991, 1996, 2001, 2006	Cross-sectional; Females; ≥19 years old; 226,169 cases	Canada; postal code	Income (Average income per single person equivalent in the enumeration area or dissemination area, categorized into quintiles)	Incidence
[47]Yost, 2001 (United States)	The California Cancer Registry 1988–1992 US Census 1990	Cross-sectional; Females; ≥15 years old; 97,227 cases; 80% white, 6% black, 9% Hispanic, 5% Asian	California; Census block group	Composite SES (Based on education index, proportion with a blue-collar job, % in workforce without a job, median household income, % below 200% poverty level, median rent, median house value, categorized into quintiles)	Incidence
[37]Palmer, 2012 (United States)	The Black Women’s Health Study, baseline in 1995 with follow-up through 2009; US Census 2000	Longitudinal; Females; 21–69 years at baseline; total n=55,896, analysis on n=1,343 cases with geocoded data; 100% black	17 US states; Census block group	Composite SES (Based on median household income, median housing value, % household receiving interest, dividends or net rental income, % with college degree, % employed in managerial, executive or professional specialty, % families with children headed by a single female; categorized into quintiles)	Incidence
[38]Panczak, 2012 (Switzerland)	The Swiss National Cohort 2001–2008;Swiss Census 2000	Longitudinal; Females; ≥ 30 years old; n=4,300,000 (including males), breast cancer deaths unknown	Switzerland; Neighborhood boundaries	Swiss-SEP Index (SES composite measure based on occupational status, income, educational attainment, family instability, persons living in low standard housing likely to have difficulty with English, categorized into deciles)	Mortality
[43]Schlichting, 2012 (United States)	SEER 2000–2007; US Census 2000	Cross-sectional; Females n=34,3627 cases; 75% white, 9% black, 9% Hispanic, 7% Asian/Pacific Islander, <1% American Indian/ Native Alaskan	17 SEER regions; County	Education (% without high school degree, categorized into quartiles) Poverty (% below federal poverty level, categorized into three groups (<10%, 20-19%, ≥20%) Urbanization (Rural–urban continuum definition per US Dept. of Agriculture, categorized into metro counties and non-metro counties)	Incidence
[36]Markossian, 2012 (United States)	SEER 1992-2007	Cross-sectional; Females; ≥15 years old; n= 23,500 cases; 69% white, 31% black	Georgia (15 counties; County	Urban/Rural residence (County-level urban/rural residence)	Mortality

### Data sources

The most common source for breast cancer data included national and state cancer registries. Of the 23 U.S.-based studies, 8 studies utilized U.S. Surveillance Epidemiology and End Results (SEER) registry data [4,16,20,28,32,35,36,43], and 9 studies utilized regional or state cancer registry data [21,31-33,39-41,46,47]; the remaining studies used data from individual research studies (two case–control [42,46], and two cohort studies [37,40]). Data for the ABR measures used in these studies were mostly from national census surveys. The majority of the U.S. studies used data from California [31,32,39-41,47], SEER regions (these include Atlanta, Connecticut, Detroit, Hawaii, Iowa, New Mexico, San-Francisco-Oakland, Seattle-Puget Sound, Utah, Los Angeles, San Jose-Monterey, rural Georgia, the Alaska Native Tumor Registry, Greater California, Kentucky, Louisiana, and New Jersey) [4,16,20,32,35,36,43], and North American Association of Central Cancer Registries (NAACCR) data [19]. Other areas included New York [21], North Carolina [18], Massachusetts [46] and Wisconsin [42]. Other U.S. studies used nationally representative survey data (National Longitudinal Mortality Study and Third National Cancer Survey [17,28]), and one nationally recruited study population [37]. All Canadian (3 studies) [27,30,35] and U.K. studies (2 studies) [22,44] used cancer registry data to identify breast cancer cases. Other studies from Australia (two cohort studies) [23,29], Italy (one study) [45] and Switzerland (one study) [38] obtained breast cancer data from individual research studies.

### Study design and sample characteristics

All studies analyzed breast cancer data in females with varying age inclusion criteria ranging from ages 15 and older to ages 70–75 years. Racial distribution of the analytic samples was not consistently reported, with only 13 studies, all based in the U.S., reporting the racial distribution of the study population [17-20,28,31,33,34,36,37,39,43,47]. Of these, one study each included only Hispanic women [31], only African-American women [37], and only white women [39]. Studies that included more than one racial group were comprised of predominantly white women (making up between 69% and 98% of the study population). Most studies (25 studies), utilized data with a cross-sectional design [4,16-23,26-28,30-36,39,41,43-45,47], two were case–control studies [42,46], and four were cohort studies [29,37,38,40]. In addition to individual-level demographic covariates such as age and race/ethnicity, 7 studies included individual-level risk factors for breast cancer such as family history of breast cancer, mammography use, parity, lactation, menarche, physical activity, alcohol intake, body mass index, hormone replacement use, oral contraceptive use and menopausal status [36-38,40,42,45,46].

### Area-Based Residential (ABR) measures

The majority of ABR measures captured different aspects of socioeconomic environment including education [16,17,39,43], income [16,17,26,27,35,39], poverty [4,19,20,30,39,43,46], summary measures of several indicators of SES (hereafter, composite SES) [22,23,31-34,37,38,41,42,44-47] and occupational class [20,39]. Income and education measures were respectively based on median family or household income, and median years of school completed or percent of the population with college or high school degree. Poverty measures included the proportion of the population living below the federally defined poverty level as determined by the annual household size adjusted income. Occupational class was assessed based on the proportion of adults employed in working class occupations [20,39]. Measures of composite SES were created using a combination of variables such as income, education, occupation, and housing characteristics. In the U.S. studies, such composite measures varied in their definitions and component variables; however, the two U.K. studies were consistent in the use of the Townsend Index of Social Deprivation, a summary residential deprivation score defined by percent of economically active residents aged 16–59 who are unemployed, percentage of private households that do not possess a car, percentage of private households that are not owner-occupied and the percentage of private households with more than one person per room [48]. Relative income was assessed as the median household income for each population decile divided by median household income of the poorest decile [35,45]. Other ABR measures included urbanization and Hispanic enclave. Urbanization was based on residence in rural versus urban areas, or metropolitan versus non-metropolitan areas, as defined by population density [18,19,21,28,29,36,40-43]. Hispanic enclave was defined as the proportion of Hispanic, Spanish speaking and linguistically isolated individuals within the area [31].

Studies of breast cancer incidence included ABR measures of education in 4 studies [16,17,39,43], income or income inequality in 6 studies [16,17,27,35,39,45], poverty in 7 studies [4,19,20,30,39,43,46], composite SES in 13 studies [22,23,31-34,37,41,42,44-47], occupational class in 2 studies [20,39], urbanization in 8 studies [18,19,21,28,40-43], and Hispanic enclave in one study [31]. Studies of breast cancer mortality included ABR measures of income in 1 study [26], poverty in 1 study [4], composite SES in 3 studies [22,23,38], and urbanization in 2 studies [29,36].

### Geographic unit

Census tract [16,17,30,32,34,35,42,45] and census block group [20,31,33,37,39,41,46,47] levels were the most common geographic unit, used in 8 studies each. County level measures were used in 6 studies [4,18,19,26,36,43], zip code or postal code in 3 studies [27,42,44], enumeration districts in 2 studies [22,35], and local government area [23] and minor civil divisions [21] in one study each. Other studies used ABR measures corresponding to distance [29], neighborhood boundaries [38], SEER regions [28,35], and one study had a priori specification of comparison counties [32]. County, census tract and block group level measures were predominant in U.S.-based studies. With the exception of census tract used in the one study in Italy, other European and Australian studies relied on postal code, enumeration districts or local government area.

### Associations between ABR measures and breast cancer incidence

**Figure 2:**
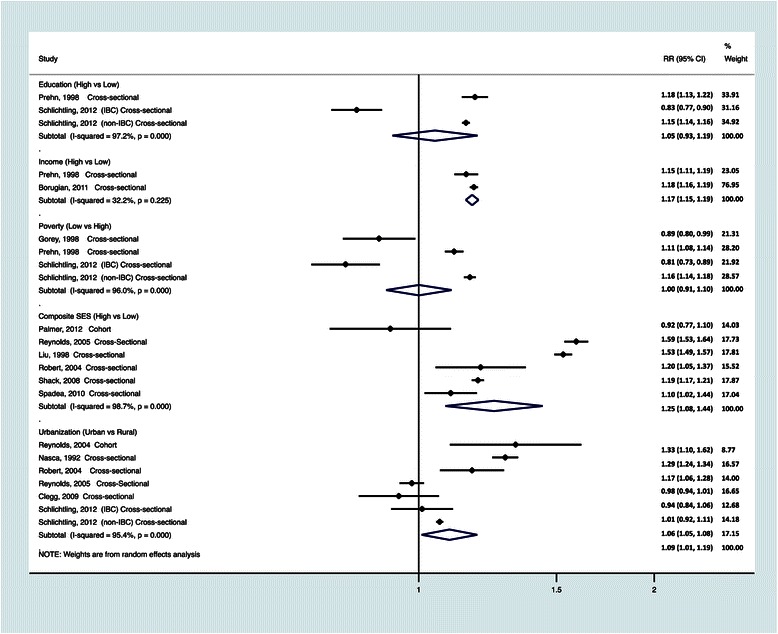
**Relative risk and 95% Confidence Interval (CI) estimates of residential environment and breast cancer incidence**^**§**^**.** The black squares and horizontal lines correspond to the study-specific relative risks and 95% confidence intervals. The area of the black squares is proportional to the inverse of the sum of the between-studies variance and the study-specific variance. The studies are organized by study design and then publication year. The diamond represents the pooled relative risk and the 95% confidence interval. ^§^Estimates were re-calculated by changing the reference category for the following studies: Income (Borugian [27]); Poverty (Gorey [30], Prehn [39]); Composite SES (Shack [44], Spadea [45]); Urbanization (Clegg [28], Schlichting [43]).

The results of associations between ABR measures and breast cancer incidence by type of ABR measure are presented in Table 3 and Figure 2, and described in the next section.

**Table 3:** **Summary of associations between residential environment and breast cancer incidence**

Author, year (location)	Main area based measure/contrast	Stratification variable	Age-adjusted rates per 100,000	Ratio measures	P-value trend
**Education**					
[17]Devesa, 1980 (United States)	Highest vs. lowest education	Whites	^a^95.9 vs. 71.9	^b^ 1.33	
		Blacks	^a^52.0 vs. 43.8	^b^1.19	
[16]Baquet, 1991 (United States)	Highest vs. lowest education	White	116 vs. 80.2	^b^ Rate Ratio: 1.45	<0.01
		Black	77.4 vs. 70.3	^b^ Rate Ratio: 1.10	0.17
[39]Prehn, 1998 (United States)	Highest vs. lowest education			^c^ Rate Ratio: 1.18 (1.13-1.22)	
[43]Schlichting, 2012 (United States)	Lowest vs. highest education	All races		^c^ Rate Ratio: IBC: 1.20 (1.12-1.30) Non-IBC: 0.87 (0.86-0.88)	
		Non-Hispanic White		^c^ Rate Ratio: IBC: 1.20 (1.09-1.32) Non-IBC: 0.96 (0.95-0.97)	
		Black		^c^ Rate Ratio: IBC: 1.28 (1.04-1.58) Non-IBC: 1.00 (0.97-1.03)	
**Income**					
[17]Devesa, 1980 (United States)	Highest vs. lowest income	Whites	^a^93.4 vs. 68.4	^b^1.37	
		Blacks	^a^48.2 vs. 47.3	^b^1.02	
[16]Baquet, 1991 (United States)	Highest vs. lowest income	White	104.3 vs. 80.7	^b^ Rate Ratio: 1.29	<0.01
		Black	108.0 vs. 67.9	^b^ Rate Ratio: 1.59	0.27
[39]Prehn, 1998 (United States)	Highest vs. lowest income			^c^ Rate Ratio: 1.15 (1.11-1.19)	
[35]Mackillop, 2000 (Canada and United States)	Highest vs. lowest income	Ontario		^c^ Rate Ratio: 1.10 (1.04-1.16)	
		US		^c^ Rate Ratio: 1.35 (1.31-1.40)	
[27]Borugian, 2011 (Canada)	Lowest vs. highest income^§^			^c^ Rate Ratio: 0.85 (0.84-0.86)	
**Poverty**					
[20]Krieger, 1990 (United States)	Lowest vs. highest poverty	Black, <40 years, High working class	11.1 vs. 9.0	^b^ Rate Ratio: 1.23	
		Black, <40 years, Low working class	18.6 vs. 13.5	^b^ Rate Ratio: 1.38	
		Black, ≥40 years, High working class	155.5 vs. 172.4	^b^ Rate Ratio: 0.90	
		Black, ≥40 years, Low working class	238.7 vs. 256.8	^b^ Rate Ratio: 0.93	
		White, <40 years, High working class	9.0 vs. 14.0	^b^ Rate Ratio: 0.64	
		White, <40 years, Low working class	9.2 vs. 5.3	^b^ Rate Ratio: 1.74	
		White, ≥40 years, High working class	214.7 vs. 209.9	^b^ Rate Ratio: 1.02	
		White, ≥40 years, Low working class	248.8 vs. 284.8	^b^ Rate Ratio: 0.87	
[30]Gorey, 1998 (Canada)	Lowest vs. highest poverty		113.23 vs. 127.65	Standardized incidence rate Ratio: 0.89 (0.80-0.99)	
[39]Prehn, 1998 (United States)	Lowest vs. highest poverty			^c^ Rate ratio: 1.11 (1.08-1.14)	
[46]Webster, 2008 (United States)	Lowest vs. highest poverty	Diagnosis year: 1990		^d^ 1.27 (0.85-1.92)	
		Diagnosis year: 1980		^d^ 0.94 (0.59-1.48)	
[4]Harper, 2009 (United States)	Highest vs. lowest poverty	Diagnosis Year: 1987	328.7 vs. 381.6	^b^ 0.86	
		Diagnosis Year: 2004	302.2 vs. 345.3	^b^ 0.88	
[19]Hausauer, 2009 (United States)	Highest vs. lowest poverty	Diagnosis Year: 2001	337.6 (326.2 – 349.2) vs. 370.4 (365.8-375.1)	^b^ 0.91	
		Diagnosis Year: 2004	305.1 (294.5-316.1) vs. 322.4 (318.2-326.6)	^b^ 0.95	
[43]Schlichting, 2012 (United States)	Highest vs. lowest poverty§	All races		^c^ Rate Ratio: IBC: 1.24 (1.12-1.37) Non-IBC: 0.86 (0.84-0.87)	
		Non-Hispanic white		^c^ Rate Ratio: IBC: 1.12 (0.99-1.27) Non-IBC: 0.87 (0.86-0.89)	
		Black		^c^ Rate Ratio: IBC: 1.32 (1.01-1.72) Non-IBC: 1.02 (0.98-1.06)	
**Composite SES**					
[23]Williams, 1991 (Australia)	Highest vs. lowest SES		203 vs. 146	^b^ Rate Ratio: 1.39	<0.001
[22]Pollock, 1997 (United Kingdom)	Highest vs. lowest SES		SIR: 105 (95–115) vs. 95 (84–107)	^b^ Standardized Incidence Rate Ratio: 1.11	
[34]Liu, 1998 (United States)	Highest vs. lowest SES			^c^ Relative Risk: 1.53 (1.49-1.57)	0.0001
[33]Krieger, 1999 (United States)	Working class poor vs. Professional	Asian and Pacific Islander		^c^ Rate Ratio: 0.8 (0.7-1.0)	0.07
		Black		^c^ Rate Ratio: 1.0 (0.9-1.1)	0.89
		Hispanic		^c^ Rate Ratio: 0.5 (0.4-0.7)	0.00
		White		^c^ Rate Ratio: 1.2 (1.1-1.3)	0.12
[47]Yost, 2001 (United States)	Highest vs. lowest SES	Whites 15–49 years		1.78 (1.7-1.9)	<0.0001
		Whites 50–64 years		1.26 (1.2-1.3)	<0.0001
		Whites 65+ years		1.21 (1.2-1.3)	<0.0001
		Blacks 15–49 years		1.70 (1.5-1.9)	0.026
		Blacks 50–64 years		1.20 (1.1-1.4)	0.008
		Blacks 65+ years		1.16 (1.0-1.3)	0.574
		Hispanics 15–49 years		2.61 (2.4-2.8)	<0.0001
		Hispanics 50–64 years		1.85 (1.7-2.0)	<0.0001
		Hispanics 65+ years		1.78 (1.7-1.9)	<0.0001
		Asian/Others 15–49 years		2.26 (2.0-2.5)	0.0001
		Asian/Others 50–64 years		1.61 (1.5-1.8)	0.0016
		Asian/Others 65+ years		1.54 (1.4-1.7)	<0.0001
[42]Robert, 2004 (United States)	Highest vs. lowest SES			^e^ Odds Ratios: 1.20 (1.05-1.37)	
[41]Reynolds, 2005 (United States)	Highest vs. lowest SES			^c^ Rate Ratio: 1.59 (1.53-1.64)	<0.01
[32]Krieger, 2006 (United States)	Highest vs. lowest SES	San Francisco Bay Area		^c^ IRR 1978–1982: 1.23 (1.10-1.38) 1988–1992: 1.40 (1.30-1.50) 1998–2002: 1.53 (1.43-1.65)	1978-1982: p=0.000; 1988–1992: p=0.000; 1998–2002 p=0.000
		San Francisco Bay Area, non-Hispanic White		^c^ IRR 1978–1982: 1.25 (1.07-1.45) 1988–1992: 0.96 (0.86-1.08) 1998–2002: 1.21 (1.06-1.38)	1978-1982: p=0.001; 1988–1992: p=0.368; 1998–2002 p=.000
		San Francisco Bay Area, Black		^c^ IRR 1978–1982: 0.82 (0.34-1.99) 1988–1992: 1.19 (0.84-1.68) 1998–2002: 0.90 (0.67-1.20)	1978-1982: p=0.159; 1988–1992: p=0.192; 1998–2002 p=0.495
		Los Angeles county		^c^ IRR 1978–1982: 1.51 (1.40-1.63) 1988–1992: 1.72 (1.64-1.81) 1998–2002: 1.79 (1.71-1.87)	1978-1982: p=0.000; 1988–1992: p=0.000; 1998–2002 p=0.000
		Los Angeles county, non-Hispanic White		^c^ IRR 1978–1982: 0.85 (0.77-0.93) 1988–1992: 1.16 (1.08-1.25) 1998–2002: 1.19 (1.11-1.26)	1978-1982: p=0.000; 1988–1992: p=0.000; 1998–2002 p=0.000
		Los Angeles county, Black		^c^ IRR 1978–1982: 1.13 (0.47-2.71) 1988–1992: 1.15 (0.82-1.60) 1998–2002: 1.21 (0.98-1.51)	1978-1982: p=0.027; 1988–1992: p=0.000; 1998–2002 p=0.003
		Massachusetts		^c^ IRR 1988–1992: 0.95 (0.88-1.02) 1998–2002: 1.35 (1.28-1.42)	1988-1992: p=0.020; 1998–2002 p=0.000
		Massachusetts, non-Hispanic White		^c^ IRR 1998–2002: 1.14 (1.07-1.21)	1998-2002: p=0.000
		Massachusetts, Black		^c^ IRR 1988–1992: 0.80 (0.35-1.83) 1998–2002: 0.68 (0.42-1.11)	1988-1992: 0=0.223; 1998–2002 p=0.911
[44]Shack, 2008 (United Kingdom)	Lowest vs. highest SES^§^			^c^ 0.84 (0.82-0.85)	
[46]Webster, 2008 (United States)	Highest vs. lowest SES	Diagnosis year: 1990		^d^ 1.30 (0.86-1.96)	
		10 years prior to diagnosis: 1980		^d^ 1.69 (1.10-2.59)	
[31]Keegan, 2010 (United States)	Highest vs. lowest SES			^c^ Rate Ratio: 1.79 (1.68-1.92)	
[45]Spadea, 2010 (Italy)	Lowest vs. highest SES^§^			^f^ 0.91 (0.84-0.98)	
[37]Palmer, 2012 (United States)	Highest vs. lowest SES			^g^ Rate Ratio: 0.92 (0.77-1.10)	0.54
**Occupational class**					
[20]Krieger, 1990 (United States)	Highest vs. lowest working class	Black, <40 years		OR: 0.57 (0.32-1.04)	
		Black, ≥40 years		OR: 0.68 (0.53-0.88)	
		White, <40 years		OR: 1.04 (0.82-1.32)	
		White, ≥40 years		OR: 0.86 (0.81-0.92)	
[39]Prehn, 1998 (United States)	Lowest vs. highest working class			^c^ Rate Ratio: 1.13 (1.09-1.17)	
**Urbanization**					
[21]Nasca, 1992 (United States)	Urban vs. rural		SIR: 107 (104–110) vs. 83 (81–86)	^b^ Standardized Incidence Rate Ratio: 1.29	<0.03
[40]Reynolds, 2004 (United States)	Urban vs. less urban			^e^ Hazard Ratio: 1.33 (1.10-1.62)	
[42]Robert, 2004 (United States)	Urban vs. rural			^e^ Odds Ratios: 1.17 (1.06-1.28)	
[18]Hall, 2005 (United States)	Metropolitan vs. non-Metropolitan non-adjacent	White	122.7 vs. 104.0	Rate Ratio: 1.18	
		Non-White	107.3 vs. 108.0	Rate Ratio: 0.99	
	Urban vs. rural	White	116.6 vs. 98.8	^b^ Rate Ratio: 1.18	
		Non-white	91.0 vs. 100.9	^b^ Rate Ratio: 0.90	
[41]Reynolds, 2005 (United States)	Urban vs. small town/rural			^c^ Rate Ratio: 0.98 (0.94-1.01)	
[28]Clegg, 2009 (United States)	Rural vs. urban ^§^		157.6 vs. 147.1	^h^ 1.06 (0.94-1.19)	
[19]Hausauer, 2009 (United States)	Urban vs. rural	Diagnosis year: 2001	375.1 (371.9-378.3) vs. 306.2 (292.9-320.1)	^b^ 1.23	
		Diagnosis year: 2004	323.5 (320.6-326.4) vs. 283.1 (270.5-295.1)	^b^ 1.14	
[43]Schlichting, 2012 (United States)	Non-metro vs. metro^§^	All races		^c^ Rate Ratio: IBC: 0.99 (0.90-1.08) Non-IBC: 0.94 (0.93-0.95)	
		Non-Hispanic white		^c^ Rate Ratio: IBC: 0.95 (0.85-1.05) Non-IBC: 0.88 (0.87-0.89)	
		Black		^c^ Rate Ratio: IBC: 1.40 (1.06-1.81) Non-IBC: 0.97 (0.92-1.01)	
**Hispanic enclave**					
[31]Keegan, 2010 (United States)	Lowest enclave vs. highest enclave			^c^ Rate Ratio: 1.79 (1.67-1.92)	
	High SES low enclave vs. low SES high enclave			^c^ Rate Ratio: 1.56 (1.50-1.63)	
**Relative index of inequality**					
[45]Spadea, 2010 (Italy)	Relative Index of Inequality			^f^ 0.92 (0.82-1.02)	
**Natural log of relative income**					
[35]Mackillop, 2000 (Canada and United States)	Natural log of relative income for regression	Ontario		^c^ Rate Ratio: 1.04 (1.00-1.08)	
		US		^c^ Rate Ratio: 1.14 (1.12-1.17)	

^a^Age and area adjusted rates.

^b^Calculated age-adjusted ratio measures for highest vs. lowest categories. Other ratio measures are presented as reported in the original article.

^c^Age adjusted rates.

^d^ Odds ratios adjusted for age, race, body mass index (BMI), alcohol use, personal and family history of breast cancer (in a mother, sister, or daughter), menstrual history, reproductive history (no children, age at first birth below or above 30 years), history of mammography (ever/never), oral contraceptive use, pharmaceutical hormone use, and exposure to ionizing radiation.

^e^ Odds ratios adjusted for age, education, mammography screening, family history of breast cancer, parity, alcohol intake/day, body mass index, age at first birth, hormone replacement, oral contraceptives, and mutually for community SES index and Urbanicity.

^f^ Adjusted for age, area of birth, education, occupational class, and housing characteristics.

^g^ Adjusted for age, time period, parity, age at first birth, lactation, age at menarche, family history of breast cancer, age at menopause, oral contraceptive use, menopausal female hormone use, body mass index, vigorous exercise, alcohol consumption, region, mammography use, and years of education.

^h^ Rate ratios adjusted for age at survey and CPS cohort.

^§^Estimates were re-calculated for meta-analysis to be consistent with the comparison of highest versus lowest ABRV.

#### Education

Of the four studies presenting results for ABR education, 3 studies reported higher incidence for the highest versus lowest ABR education [16,17,39]. The fourth study examined inflammatory breast cancer (IBC) and non-IBC separately, [43] and reported higher incidence of IBC in the lowest vs. highest ABR education, but consistent with positive associations between education and overall breast cancer, found lower non-IBC incidence in the lowest vs. highest ABR education. Three studies reported estimates of the association between ABR education and incidence for blacks and whites [16,17,43]. The associations appeared stronger in whites than in blacks in two studies (RR = 1.33 and 1.45 among whites, 1.10 and 1.19 among blacks) [16,17]; and the association was only statistically significant in white women in the study that reported statistical significance of the estimates [16]. Similarly, the remaining study reported slightly lower non-IBC incidence in the lowest versus highest ABR education in whites, (RR = 0.96, 95% CI: 0.95-0.97) but found no significant ABR education differences in non-IBC risk for blacks (RR = 1.00, 95% CI: 0.97-1.03); however, for the rare IBC sub-type, incidence was higher in the lowest versus highest ABR education in both whites (RR = 1.20, 95% CI: 1.09-1.32) and blacks (RR = 1.28, 95% CI: 1.04-1.58) [43].

#### Meta analysis- education

Two studies met the criteria for inclusion in the meta-analysis, shown in Figure 2 [39,43], with one study presenting results for two subtypes of IBC and non-IBC. The meta-analysis summary RRs for the highest versus lowest ABR education was 1.05 (95% CI: 0.93, 1.19). There was evidence of statistically significant heterogeneity between the studies (P <0.01; I^2^ = 97.2%), although there was no evidence of small-study bias as confirmed with Egger’s test (p = 0.25). Influence analysis indicated that the meta-analysis results were dominated by the non-IBC results presented by Schlichting et al., and excluding this study would have attenuated the observed associations. These two studies were the only studies eligible for meta-analysis that also presented race-stratified estimates for blacks and whites. The results of meta-analysis using the race-stratified data yielded similar estimates with summary RR for the highest versus lowest ABR education of 1.05 (95% CI: 0.87-1.27) among whites, and summary RR of 1.00 (95% CI: 0.85-1.18) among blacks (data not shown).

#### Income

Five studies examined ABR income and incidence, and all reported higher incidence with higher ABR income [16,17,27,35,39]. The 2 studies with un-stratified estimates reported a statistically significant 15% higher breast cancer incidence associated with residence in higher ABR income areas [27,39]. Two studies reported estimates for whites and blacks, with both studies showing higher incidence for the highest versus lowest ABR income in whites (RRs of 1.37 and 1.29) [16,17], and one study showing the same association in blacks (RR = 1.59) [16]; one study also reported a statistically significant p-value for linear trend across categories of income in whites only [16]. One study evaluated ABR income and incidence in Ontario, Canada and in the U.S. [35], and reported positive and statistically significant association in both Canada (RR = 1.10, 95% CI: 1.04-1.16) and the U.S. (RR = 1.35, 95% CI: 1.31-1.40), with stronger associations in the U.S.

#### Meta analysis- income

Two studies met the criteria for inclusion in the meta-analysis [27,39]. The meta-analysis summary RRs for the highest versus lowest ABR income was 1.17 (95% CI: 1.15, 1.19). There was slight evidence of significant heterogeneity between the studies (P = 0.23; I^2^ = 32.2%), and no evidence of small-study bias as confirmed with Egger’s test (p = 0.25).

#### Poverty

Seven studies presented results on ABR poverty [4,19,20,30,39,43,46], of which three studies reported un-stratified results [30,39,43]. Of these 3 studies, one reported lower breast cancer incidence in the lowest versus highest ABR poverty areas (SIRR = 0.89, 95% CI: 0.80-0.99) [30], while another study observed the reverse for the same comparison (RR = 1.11, 95% CI: 1.08-1.14) [39]. The remaining study reported higher incidence for IBC (RR = 1.24, 95% CI: 1.12-1.37) and lower incidence for non-IBC (RR = 0.86, 95% CI: 0.84-0.87) in the highest versus lowest ABR poverty areas [43]. Four other studies reported stratified results for ABR poverty and incidence [4,19,20,30]. One reported estimates across three levels of stratification by race, age and occupational class [20]. In black women less than 40 years old, lowest vs. highest ABR poverty was associated with higher breast cancer incidence, regardless of ABR occupation class (RR for lowest vs. highest ABR poverty in high working class: 1.23, RR for lowest vs. highest ABR poverty in low working class: 1.38) whereas for white women less than 40 years old, lowest vs. highest ABR poverty was associated with higher incidence in low working class areas (RR for lowest vs. highest ABR poverty in low working class: 1.74) and associated with lower incidence in high working class areas (RR for lowest vs. highest ABR poverty in high working class: 0.64). In both white and black women over the age of 40, lowest versus highest ABR poverty showed lower incidence or no differences in incidence. The remaining three studies reported results stratified by year of diagnosis [4,19,46]. Two studies that compared highest to lowest ABR poverty showed lower incidence in both years of studies considered [4,19]. The last study showed mixed and non-significant results across the two diagnosis years [46]. One study reported race-stratified results for IBC and non-IBC, and showed significantly lower non-IBC incidence in highest versus lowest ABR poverty areas for whites (RR = 0.87, 95% CI: 0.86-0.89), non-significant results for blacks (RR =1.02, 95% CI: 0.98-1.06), and non-significant associations for IBC in whites (RR = 1.12, 95% CI: 0.99-1.27), but higher IBC incidence in blacks (RR = 1.32, 95% CI: 1.01-1.72) ^33^.

#### Meta-analysis- poverty

Three studies met the criteria for inclusion in the meta-analysis [30,39,43]. For consistency with other SES indicators (i.e. high SES vs. low SES), we compared low vs. high poverty areas as lower poverty areas represent higher SES areas. The meta-analysis summary RR for the lowest versus highest ABR poverty was 1.00 (95% CI: 0.91-1.10). There was evidence of significant heterogeneity between the studies (P <0.01; I^2^ = 96.0%), but no evidence of small-study bias as confirmed with Egger’s test (P = 0.86). Influence analysis indicated that the meta-analysis results were dominated by the non-IBC results presented by Schlichting et al., and excluding this study would have attenuated the observed associations.

#### Composite SES

Thirteen studies presented results on ABR composite SES [22,23,31-34,37,41,42,44-47]. Of the 9 studies that reported un-stratified estimates [22,23,31,34,37,41,42,44,45], 7 reported higher incidence in the highest versus lowest ABR composite SES [23,31,34,41,42,44,45], one showed a non-significant higher incidence [22], and one showed a non-significant lower incidence [37]. Two studies provided race-stratified estimates; one study used a combination of area based working class status and poverty as a measure of composite SES, and observed higher incidence among working class poor versus professionals whites (RR = 1.2, 95% CI: 1.1-1.3), lower incidence among working class poor versus professionals Hispanics (RR = 0.5, 95% CI: 0.4-0.7), and non-significant differences in Asian and Pacific Islanders or blacks [33]. The other study reported race- and age-stratified estimates comparing the highest versus lowest ABR composite SES and showed significantly higher incidence in all age groups among whites, blacks, Hispanics and Asian/Others [47]. One study reported estimates stratified by region and time period [32]. In the San Francisco Bay Area, breast cancer incidence was higher for the highest versus lowest ABR composite SES in all three time periods, with rates increasing significantly from 1.23 (95% CI: 1.10-1.38) in 1978–1982 to 1.53 (95% CI: 1.43-1.65) in 1998–2002. The higher incidence associated with higher composite SES was observed for white women in 1978–1982 (RR = 1.25, 95% CI: 1.07-1.45) and 1998–2002 (RR = 1.21, 95% CI: 1.06-1.38), but not for black women in any of the time periods. Similar patterns of higher breast cancer incidence in higher SES areas were observed in the same time period in Los Angeles County, and in1988–1992 and 1998–2002 in Massachusetts for the overall sample and white women, but these were not statistically significant for black women. Another study reported estimates stratified by diagnosis year, and showed higher but non-significant incidence for the highest versus lowest ABR composite SES in 1990 (RR = 1.30, 0.86-1.96), and significantly higher incidence for the same contrast in 1980 (RR = 1.69, 95% CI: 1.10-2.59) [46].

#### Meta-analysis- composite SES

Seven studies met the criteria for inclusion in the meta-analysis [31,34,37,41,42,44,45]. The studies by Keegan et al. [31] and Reynolds et al. [41] both presented results for residential composite SES based on data from the same study population with overlap in the study period. We included the study by Reynolds in the meta-analysis since it had a larger sample size. The meta-analysis summary RR for the highest versus lowest residential composite SES for the six studies included in the meta-analysis was 1.25 (95% CI: 1.08-1.44). There was evidence of significant heterogeneity between the studies (P<0.01, I^2^=98.7%), but no evidence of small-study bias as confirmed with Egger’s test (P=0.74). We performed meta-regression analysis to identify potential sources of heterogeneity in the meta-analysis results adjusting for study year, study design (cross-sectional versus cohort) and adjustment for other covariates in the analysis. However, we found no evidence that these factors contributed to the observed heterogeneity in this meta-analysis. None of the variables were statistically significant in the analysis, and the between-study variance changed very slightly from 0.03 to 0.033 after adjusting for the covariates. Influence analysis indicated that the meta-analysis results were dominated by the results presented by Shack et al. [44] and Palmer et al. [37]. Excluding the Shack et al. study strengthened the observed association, while excluding the Palmer et al. study attenuated the observed associations.

#### Occupational class

Two studies presented results on ABR occupational class [20,39], showing mostly lower incidence in the highest versus lowest ABR working class. One study showed higher breast cancer incidence in the lowest versus highest ABR working class (RR=1.13, 95% CI: 1.09-1.17) [39]. The other study showed that among black women of all ages and among white women ages 40 years and older, breast cancer incidence was lower in highest versus lowest ABR working class (ORs ranging between 0.57-0.86), but no differences by ABR occupational class were observed for white women less than 40 years (OR=1.04, 95% CI: 0.82-1.32) [20]. A summary estimate was not provided, as there were too few studies meeting the eligibility criteria for a meta-analysis.

#### Urbanization

Eight studies presented results on urbanization and incidence [18,19,21,28,40-43]. Six studies reported un-stratified results [21,28,40-43], and 4 of these showed significantly higher breast cancer incidence ranging from 14%-33% in urban versus rural areas [19,21,40,42]. One study reported results for IBC and non-IBC; residing in a non-metropolitan versus metropolitan areas was associated with lower incidence of non-IBC (RR=0.94, 95% CI: 0.93-0.95), while the association for IBC was not statistically significant [43]. Two studies presented estimates of the association between urbanization and incidence stratified by race [18,43]. In one study, incidence was higher in white women in metropolitan (RR=1.18 relative to non-metropolitan area) and urban areas (RR=1.18 relative to rural area), but no statistical test results were reported; results were less clear for nonwhite women [18]. In the other study [43], similar associations were observed for non-IBC, with incidence being lower among white women in non-metropolitan compared with metropolitan areas (RR= 0.88, 95% CI: 0.87, 0.89), but differences were not statistically significant among black women (RR=0.97, 95% CI: 0.92, 1.01). In contrast, IBC incidence among white women was not significantly different across metropolitan and non-metropolitan areas, but was significantly higher among black women in non-metropolitan versus metropolitan areas (RR=1.40, 95% CI: 1.06, 1.81). Higher incidence was also reported in urban versus rural areas for diagnosis years 2001 (RR=1.23) and 2004 (RR=1.14) in another study [19].

#### Meta-analysis- urbanization

Six studies met the criteria for inclusion in the meta-analysis [21,28,40-43]. The meta-analysis summary RR comparing urban versus rural residence was 1.09 (95% CI: 1.01-1.19). There was evidence of significant heterogeneity between the studies (P<0.01, I^2^=95.4%), but no evidence of small-study bias as confirmed with Egger’s test (P=0.80). Influence analysis indicated that the meta-analysis results were dominated by the IBC results presented by Schlichting et al., and excluding this study would have strengthened the observed associations. We performed meta-regression analysis to identify potential sources of heterogeneity in the meta-analysis results adjusting for study year, study design (cross-sectional versus cohort) and adjustment for other covariates in the analysis. However, we found no evidence that these factors contributed to the observed heterogeneity in this meta-analysis. None of the variables were statistically significant in the analysis, and the between-study variance changed very slightly from 0.01 to 0.006.

#### Hispanic enclave

One study presented results on ABR Hispanic enclave, and reported significantly higher incidence in lowest versus highest ABR Hispanic enclaves (RR=1.79, 95% CI: 1.67-1.92) [31]. Higher incidence was also observed in highest SES, low Hispanic enclave areas versus lowest SES, high enclave areas (RR=1.56, 95% CI: 1.50-1.63).

#### Other ABR measures

One study computed a relative index of inequality, and showed a non-significant lower incidence with increasing ABR inequality (RR=0.92, 95% CI: 0.82-1.02)[45]. Another study used the natural log of relative income in Ontario and the US [35], showing borderline statistically significant higher incidence with higher relative income in both countries (Ontario RR=1.04, 95% CI: 1.00-1.08; US RR=1.14, 95% CI: 1.12-1.17).

### Associations between ABR measures and breast cancer mortality

The results of the associations between ABR measures and breast cancer mortality by type of ABR measure are presented in Table 4 and described in detail below.

**Table 4:** **Summary of associations between residential environment and breast cancer mortality**

Author, year (location)	Main area based measure/contrast	Stratification variable	Age-adjusted rates per 100,000	Ratio measures	P-value Trend
**Income**					
[26]Blot, 1977 (United States)	High income vs. low income	Northeast, <10,000	^a^ 25.2 vs. 24.1		
		South, <10,000	^a^ 18.0 vs. 16.2	^b^ Rate Ratio: 1.05	
		Central, <10,000	^a^ 22.0 vs. 20.9	^b^ Rate Ratio: 1.11	
		West, <10,000	^a^ 20.7 vs. 20.3	^b^ Rate Ratio: 1.05	
		Northeast, 250,000+	^a^ 30.2 vs.28.1	^b^ Rate Ratio: 0.99	
		South, 250,000+	^a^ 25.1 vs. 22.9	^b^ Rate Ratio: 1.07	
		Central, 250,000+	^a^ 28.6 vs. 26.4	^b^ Rate Ratio: 1.08	
		West, 250,000+	^a^ 26.8 vs. 23.6	^b^ Rate Ratio: 1.14	
**Poverty**					
[4]Harper, 2009 (United States)	Highest vs. lowest SES	Year of death: 1987	85.1 vs. 102.7	^b^ 0.83	
		Year of death: 2004	76.3 vs. 74.4	^b^ 1.02	
**Composite SES**					
[23]Williams, 1991 (Australia)	Highest vs. lowest SES		^a^ 68 vs. 57	^b^ Rate Ratio: 1.19	
[22]Pollock, 1997 (United Kingdom)	Highest vs. lowest SES		SMR: 99 (84–116) vs. 111 (93–132)	^b^ Rate Ratio: 0.89	
[38]Panczak, 2012 (Switzerland)	Lowest vs. highest SES			^c^ Hazard Ratio: 0.96 (0.87-1.05)	0.826
**Urbanization**					
[29]Dobson, 2010 (Australia)	Remote vs. major urban centers			^d^ Hazard Ratio: 0.47 (0.06-3.42)	
[36]Markossian, 2012 (United States)	Rural vs. urban			^e^ Hazard Ratio: 1.04 (0.85-1.26)	0.748

^a^ Age adjusted rates.

^b^ Calculated age-adjusted ratio measures for highest vs. lowest categories. Other ratio measures are presented as reported in the original article.

^c^ Adjusted for age, sex, nationality, marital status, level of urbanization, individual-level education and professional status.

^d^ Age-adjusted hazard ratio.

^e^ Adjusted for race, tumor stage, tumor grade, hormone receptor status and treatment (surgery/radiation).

#### Income

One study presented results on ABR income, stratified by country region and population size [26]. Mortality was higher by 5-14% in the highest versus lowest ABR income in all the strata, with the exception of U.S. Northeast areas with population size > 250,000 (RR=0.99); however, no data on statistical tests was presented.

#### Poverty

One study examined ABR poverty and breast cancer mortality. Results were stratified by year of death [4], and reported lower mortality for highest versus lowest ABR poverty in 1987 (RR=0.83), and estimates approaching null in 2004 (RR=1.02), but provided no data on statistical tests.

#### Composite SES

Three studies presented results on ABR composite SES [22,23,38]. Two studies showed higher mortality in highest versus lowest ABR composite SES, however one study did not provide data on statistical significance [23], and the other study was not statistically significant [22]. The third study reported lower mortality among residents of high ABR composite SES areas and the results were also not statistically significant [38].

#### Urbanization

Two studies presented results on urbanization, comparing rural or remote areas with urban areas [29,36]. One study reported non-significant lower mortality in remote areas (Hazard Ratio: 0.47, 95% CI: 0.06-3.42) [29]. The second study reported a non-significant higher mortality (Hazard Ratio: 1.04, 95% CI: 0.85-1.26) [36] in rural areas relative to urban areas with non-significant p-value for trend across levels of urbanization.

#### Meta-analysis

Two studies met the criteria for inclusion in the meta-analysis (figure not shown) [29,36]. The meta-analysis summary RR comparing urban versus rural residence was 0.97 (95% CI: 0.79-1.18). There was no evidence of significant heterogeneity between the studies (P=0.44, I^2^=0.0%), and no evidence of small-study bias as confirmed with Egger’s test (P=0.62).

## Discussion

This review and meta-analysis summarizes and quantifies the literature examining the association between a variety of constructs representing the residential environment and breast cancer incidence and mortality. There has been a substantial increase in the number of studies assessing the influence of the residential environment in relation to breast cancer in the past decade; over 30% of all studies on this topic were published in the past 3 years. Overall, residing in urban areas and in higher socioeconomic areas characterized by higher income, and composite SES was associated with higher breast cancer incidence. Specifically, the results from articles included in the meta-analysis showed that urban residence was associated with a borderline significant 9% increase in breast cancer incidence, and higher income and higher composite SES were respectively associated with a significant 17% and 25% increase in breast cancer incidence. The meta-analysis results for the association between higher education and lower poverty and increased breast cancer incidence appeared to have been attenuated due to the inverse associations between these variables and IBC incidence, a relatively rare type of cancer accounting for <6% of all breast cancers diagnosed [49], and excluding IBC data from the meta-analysis resulted in statistically significant associations between higher breast cancer incidence and higher education and lower poverty. The associations between the residential environment and breast cancer mortality were less consistent across studies, with different studies showing both higher and lower mortality in urban vs. rural areas and in areas with high vs. low composite SES. We observed significant heterogeneity between studies included in both the systematic review as well as in the meta-analysis, which may be driven by differences in study design, construction of area level measures, and geographic units. Due to the observed heterogeneity between studies on breast cancer incidence, and the lack of statistical significance reporting in most of the studies on breast cancer mortality, the overall results should be interpreted with caution.

The positive association between ABR composite SES and breast cancer incidence was the strongest and most consistent association observed in this review. There is no clear consensus on the use of single SES indicators such as income or education versus summary or composite SES measures to characterize associations between socioeconomic status and health outcomes [50,51]. Both types of measures have been used extensively, and the decision regarding which type of measure to use should be driven by the research question of interest and hypothesized causal pathways. Measures of single SES indicators are often only modestly correlated, and may show associations of varying magnitude or direction with the same health outcomes [52]. In such cases, the use of a composite SES measure may result in null associations whereas the use of single SES indicators may provide insight into plausible pathways through which specific SES factors influence health. However, in the context of residential environment, the relationship between SES and health may not be driven solely by income, education or poverty per se, but instead may reflect exposure to complex and environments that are better captured through the use of a composite measure of SES.

Although our selection criteria did not specify geographic region, the majority of studies included in this review were conducted in the U.S., and thus the results may be more generalizable to the U.S. population. The positive association between residential composite SES and breast cancer incidence in the U.S. was also observed in studies from Australia, Canada and the U.K. The increase in breast cancer incidence associated with highest compared with lowest composite SES ranged from 20% in the U.K. and Canada to 40% in Australia. The similar associations observed in these countries with more inclusive social welfare and healthcare systems than those present in the U.S., including national healthcare insurance systems, which provide everyone with some level of access to healthcare, suggest the possibility of other pathways linking residential environment to breast cancer risk [53].

Potential pathways linking the residential environment with breast cancer risk or mortality include: physical attributes of an area that may promote or hinder breast cancer risk factors such as reproductive factors, diet and physical activity [7,54,55]; availability of resources relevant for screening and diagnoses such as access to mammogram facilities and clinics [10,11]; or psychosocial pathways involving stress and anxiety due to residential crime and safety [56,57]. The influence of these area-level factors on breast cancer risk or mortality may be independent of individual-level factors including social status or health insurance, and the importance of investigating the potential mediating effect of individual-level factors in understanding how the residential environment operates in shaping health outcomes have previously been highlighted [58,59]. However, this topic remains largely unexplored in the literature included in this review as only 5 of the articles examined individual-level breast cancer risk factors status [37,40,42,45,46], and only 4 studies examined individual-level SES [37,40,42,45]. Of the 3 studies that controlled for an individual-level measure of SES in addition to an ABR SES measure [37,42,45], two studies reported a 10-20% increase in breast cancer risk in the highest versus lowest composite SES areas after adjustment for individual-level educational status [42,45], and one study, conducted only in black women, observed no significant association [37]. Overall, it is difficult to draw any conclusions about mechanisms or pathways of ABR influences on breast cancer outcomes, as most of the studies included in the current review did not explicitly hypothesize or test a specific pathway through which the residential environment could influence breast cancer in women. As suggested by other reviews, many studies include area level constructs mainly as a proxy for missing individual-level factors that are known to influence breast cancer risk and mortality, which limit their interpretation in terms of residential or area level influences on breast cancer outcomes [60,61].

With the exception of a few U.S. studies, the reviewed studies included populations comprised of predominantly white women. Several U.S. studies that presented results separately for black women or included substantial number of black women reported inconsistent and mostly non-significant associations in black women. More research is needed to understand whether the associations between residential environment and breast cancer are consistent across U.S. racial/ethnic populations. Research in other countries with racial/ethnic diversity may also benefit from examining racial differences in the associations of ABR constructs and breast cancer outcomes as these may be different from those observed in the U.S.; however, availability of data on race/ethnicity in cancer surveillance data may be limited in certain countries.

The majority of reviewed studies employed a cross-sectional design. For example, in most studies, breast cancer outcome and the residential environment construct were assessed contemporaneously, that is, there was no consideration given to the potential induction period between the exposure and diagnosis of breast cancer. The majority of the studies used administrative or secondary data sources (e.g. Census surveys) in developing ABR measures, while cancer registries (e.g. SEER) were the most common source of breast cancer data. The use of administrative data for ABR measures raises the issue of potential measurement error, since these databases were not designed for research purposes. There is also the issue of constantly changing geographic boundaries, which may result in misclassification if such changes are differential with respect to SES or other ABR measures. Finally, very few studies conducted multi-level analysis to account for the hierarchical nature of the data with individuals nested within neighborhoods. Further studies in this area should take advantage of the growing arsenal of methods available to deal with complex, multilevel studies such as structural equation models, Bayesian methods, and agent-based models.

There are several limitations in this review. The included studies were restricted to peer-reviewed, published, English language articles. The English language may have resulted in selection of large number of studies conducted in predominantly English-speaking countries including the U.S., Canada, U.K. and Australia. By restricting the search to studies that focused on breast cancer specifically, we may have missed studies with a more general title of ‘cancer’ during title review. However, we also searched the references of included articles, minimizing the likelihood of missing a significant number of studies. The review criteria resulted in the exclusion of articles that were focused on physical environmental exposures. We believed that the examination of physical environmental exposures (e.g. proximity to power lines) in relation to breast cancer constituted a separate class of exposures. As demonstrated in this review, studies included in the meta-analysis examined different ABR constructs and measurement. In addition to the heterogeneity in the exposure definition, individual studies examined different contrasts (e.g., quintiles, quartiles) and the same exposure contrast was not examined across the individual studies. We chose to include the most extreme contrast for this meta-analysis (e.g., highest vs. lowest category for comparison), which is standard practice in conducting a meta-analysis. However, the pooling of these contrasts could contribute to some of the between-studies heterogeneity that was observed for education, poverty, composite SES and urbanization, and it prevents us from examining the dose–response relationship between those exposures and the outcome. To identify potential sources of heterogeneity in the meta-analysis results based on study characteristics, we performed meta-regression analysis; we observed no statistically significant evidence that factors such as year of publication, study design and multivariable adjustment contributed to the observed heterogeneity in this meta-analysis. Standardization of ABR measures across future studies should help to improve synthesis of findings and our understanding of residential environment influences on breast cancer outcomes.

There are also several strengths in this review. We used broad search criteria to improve our ability to select studies with a wide range of ABR measures, which may have been labeled differently across studies. In addition, the use of multiple ABR measures allowed us to examine relationships between different ABR constructs in relation to breast cancer outcomes. The meta-analysis of similar studies allowed us to generate summary estimates of the magnitude of the association, information that has been lacking in this field. Although there was significant heterogeneity between studies within ABR constructs, the summary estimates provide a useful overview and highlights some gaps in the current published literature. This review imposed no restriction on geographic location of published studies. This is important to highlight differences and similarities in studies across countries, which may provide important information about potential effect modifiers (e.g. access to healthcare resources).

## Conclusions

In conclusion, this is the first systematic review and meta-analysis of studies examining the associations between ABR constructs (income, education, poverty, composite SES, occupational class and urbanization) and breast cancer incidence and mortality. Lack of *a priori* specification of a conceptual model linking the residential environment with individual outcomes, inadequate considerations of temporality, inconsistencies in the geographic unit, lack of proper consideration of potential confounders and mediators, and lack of multi-level analytic techniques have all been identified as limitations in this field [58,60,61], and were the same limitations in most of the studies included in this review. Some of these limitations were further reflected in the statistically significant test for heterogeneity even between studies examining the same construct. While these conceptual and methodological issues limit our ability to draw definitive conclusions, we observed modest positive associations between breast cancer incidence residential area environment, as measured by ABR income and composite SES, and urbanization. Data from current studies did not allow for proper assessment of ABR measures in non-white women, but the limited data available for black women suggest less consistent associations between ABR measures and breast cancer incidence in black women. It is unclear whether the observed associations would remain once appropriate multi-level analytic methods with proper control for confounders are employed.
